# The Statistical Significance of Suffering

**DOI:** 10.1371/journal.pmed.0020421

**Published:** 2005-12-27

**Authors:** Kristen Suthers

**Affiliations:** **1**GDNF 4 Parkinson'sWashington, District of ColumbiaUnited States of America

Musa Mayer makes several good points about the importance of enrolling people with life-threatening conditions in clinical trials in order to identify new treatments and speed the pipeline along for the greater good [1]. However, the idea that clinical trial enrollment suffers when seriously ill individuals are provided compassionate use of treatments is myopic; one does not negate the other. In many cases, persons who seek compassionate use of medications are ineligible for the clinical trials Mayer would want them to enroll in, and will likely die or suffer considerably before the experimental treatment they are seeking is approved for the public. In a world of limited resources, we need to ask, how do we encourage enrollment in clinical trials to develop treatments and cures that will benefit people in the future, while humanely treating those who are ineligible for these trials and suffer right now? The first step is to understand that clinical trial enrollment and compassionate-use programs are not competing interests today, as they perhaps were in the 1980s and 1990s. The next step is to educate the public, not only about the importance of enrollment in clinical trials, but about their rights as informed participants in the noble process of science. Mayer's perspective [1] fails to consider the ultimate goal of clinical trials: to relieve human suffering. It serves no one's interest to demand an all-or-nothing approach to scientific progress. As Einstein said, “Not everything that can be counted counts, and not everything that counts can be counted.”
